# Terrestrial isopods as model organisms in soil ecotoxicology: a review

**DOI:** 10.3897/zookeys.801.21970

**Published:** 2018-12-03

**Authors:** Cornelis A.M. van Gestel, Susana Loureiro, Primož idar

**Affiliations:** 1 Department of Ecological Science, Faculty of Science, Vrije Universiteit, De Boelelaan 1085, 1081 HV Amsterdam, The Netherlands Vrije University Amsterdam Netherlands; 2 University of Aveiro, Department of Biology and the Centre for Environmental and Marine Studies, Campus Universitário de Santiago, 3810-193, Aveiro, Portugal University of Aveiro Aveiro Portugal; 3 Department of Biology, Biotechnical Faculty, University of Ljubljana, Večna pot 111, SI-1000 Ljubljana, Slovenia University of Ljubljana Ljubljana Slovenia

**Keywords:** Bioaccumulation, biomonitoring, indicator organisms, Isopoda, toxicity tests

## Abstract

Isopods play an important role in the decomposition of leaf litter and therefore are making a significant contribution to nutrient cycling and soil ecosystem services. As a consequence, isopods are relevant models in soil ecotoxicology, both in laboratory toxicity tests and in field monitoring and bioindication studies. This paper aims at reviewing the use of isopods as test organisms in soil ecotoxicology. It provides an overview of the use of isopods in laboratory toxicity tests, with special focus on comparing different exposure methods, test durations, and ecotoxicological endpoints. A brief overview of toxicity data suggests that chemicals are more toxic to isopods when exposed through soil compared to food. The potential of isopods to be used in bioindication and biomonitoring is discussed. Based on the overview of toxicity data and test methods, recommendations are given for the use of isopods in standardized laboratory toxicity tests as well as in situ monitoring studies.

## Introduction

Increasing human activities have caused serious effects on man and the environment. Since the industrial revolution in the 19^th^ century, pollution from industries and metal contamination from mining activities has increased. In the 20^th^ century, after the Second World War, the massive use of pesticides resulted in widespread environmental pollution. The first evidence of chemical pollution was shown by air pollution, for instance leading to smog episodes in cities. Air pollution was a major problem already in the 19^th^ century and still is one of the major factors threatening human health, especially in rapidly developing industrial regions like China (see e.g., Kan et al. 2012). The quality of surface waters also became seriously affected, with many incidences of fish killings all over the world (see e.g., Langer 1964); but reports on soil pollution for some time seemed to lag behind those on air and water pollution. Since the 1980s, however, soil pollution has also become highlighted as a major issue. It was realised that polluted soils are generally more difficult and considerably more expensive to remediate than cleaning or preventing water and air pollution (see e.g., Swartjes 2011).

Apart from threatening human health, air, water and soil pollution may also affect ecosystems. By affecting major functions of natural systems, pollution can alter the so-called ecosystem services (Hunt and Wall 2002; Faber and Van Wensem 2012), which include 1. provisioning services: material outputs from ecosystems, such as food, water, and other resources, 2. regulating services: ecosystems acting as regulators e.g., of the quality of air and soil or by providing flood and disease control, 3. habitat or supporting services: providing living space for plants or animals, maintaining a diversity of different breeds of plants and animals, etc., and 4. cultural services: non-material benefits people obtain from contact with ecosystems, such as aesthetic, spiritual and psychological benefits (The Millennium Ecosystem Assessment 2005; TEEB 2010). For maintaining ecosystem services, a high biodiversity is essential, as was recently shown by Soliveres et al. (2016).

Pollution may damage ecosystems and by that the support ecosystems provide to (the quality of) human life. Protection of ecosystem services therefore is essential not only for safeguarding the health of our ecosystems but also for our own benefit (Faber and Van Wensem 2012; TEEB 2010), and should thus receive more attention in the risk assessment of chemicals (see e.g., Nienstedt et al. 2012; Forbes and Calow 2013; Maltby 2013). For that reason, ecotoxicologists and ecologists are aiming at protecting ecosystems, including all key-organisms that together contribute to its functioning.

Isopods play an important role in the functioning of soil ecosystems and therefore also in the ecosystem services provided by soils. They act mainly on the first processes of litter fragmentation, contributing to the input of high quality organic matter, and increasing the microbiome for further nutrient cycling in soil (Hassall et al. 1987). This paper will discuss the possible role of isopods as model organisms in ecotoxicology. For that purpose, before focusing on isopods, we will first briefly describe some ecotoxicological concepts and the general principles of ecotoxicological risk assessment. Next we will discuss the use of isopods as model organisms in predictive (and diagnostic) ecotoxicological approaches. This will include the use of different endpoints, the relevance of different routes of exposure and the importance of bioavailability, and will be supported by a review of the literature on the toxicity and bioaccumulation of different chemicals in isopods. Finally we will discuss the use of isopods as diagnostic tools in monitoring soil quality.

## Ecotoxicology and ecotoxicological risk assessment

After the publication of the book Silent Spring by Rachel Carson (1962), awareness of the side effects of pesticides and other chemicals on the environment increased. The term ecotoxicology was coined only in 1977 by Truhaut. The aim of ecotoxicology is to protect ecosystems from adverse effects of chemical pollution, alone or in combination with other anthropogenic stressors. To realise its aims, ecotoxicology makes use of knowledge from different disciplines, like environmental chemistry, toxicology, and ecology (Van Leeuwen and Vermeire 2007).

Environmental chemistry provides insight into the way chemicals interact with components of the environment, determining their fate, and therefore also the exposure of organisms and ecosystems. From knowledge of persistence and partitioning, environmental chemistry enables estimating Predicted Environmental Concentrations (PEC), which are the starting point for the risk assessment of chemicals. One important issue is bioavailability: the notion that total concentration of a chemical in the environment is not indicative of its risk, because only a fraction of it may be available for uptake and therefore causing effects (Van Gestel 2008, 2012). Especially in soils, due to their large capacity of complexing chemicals, bioavailability is an important issue. Therefore, and besides the distribution of chemicals in environmental compartments, bioavailability will focus also on the fate of chemicals in the organisms, bridging the gap between what is in the environment and the compartmentalisation of chemicals inside biota (external versus internal bioavailability) (Vijver et al. 2004).

Toxicology provides insight into the interaction of chemical pollutants with molecules, tissues, and organs resulting in effects at the molecular and individual level (Van Leeuwen and Vermeire 2007). From (laboratory) tests, dose-response relationships are derived to provide quantitative measures of the toxicity of chemicals, like LC_50_ (lethal concentration killing 50 % of the tested population), EC_10_ or EC_50_ (concentrations causing 10 % or 50 % reduction in a measured parameter, e.g., growth or reproduction, compared with the response in the untreated control) or NOEC (No-Observed Effect Concentration, the highest concentration tested causing no significant reduction in a measured parameter compared to the untreated control). LC_50_ values usually are determined in short-term or acute toxicity tests, with mortality as the main endpoint. EC_10_, EC_50_, and NOEC values are derived in sublethal toxicity tests, using growth, reproduction or another sublethal endpoint. Such tests usually require longer exposure times, and are therefore sometimes indicated as chronic tests. The term chronic however, should only be used when test duration includes a substantial part of the life span of the test organism. From the available toxicity data, a safe concentration of a pollutant can be derived, as a so-called Predicted No-Effect Concentration (PNEC). Different approaches may be followed to derive a PNEC, extrapolating effects from acute (LC_50_) to sublethal effects (EC_10_, EC_50_ or NOEC), from one or few species to many species and from the laboratory to the field. The resulting PNEC should be protective of effects not only at the individual level but at higher levels of biological organisation, like populations and communities.

Ecology provides the knowledge for the extrapolation from individual-level effects to effects on populations, communities, and ecosystems. Ecology adds knowledge about life histories of organisms, their functioning in different processes or their interactions with other species or the abiotic environment. Knowledge on life histories and other ecological properties or traits of species also helps understanding how organisms will be exposed to chemicals in the environment and how this may affect populations. The behaviour of an organism may for instance differ depending on its life stage, while it may have great influence on exposure. Again, this seems even more important in soils, where pollution is only rarely distributed homogenously. Finally, knowledge about ecological interactions between species may help translating effects to the community and ecosystem level.

Ecotoxicology may follow predictive/prospective and diagnostic/retrospective approaches (Van Gestel 2012). Predictive approaches aim at preventing possible effects of chemicals before they are introduced onto the market or to limit side effects after introduction. Predictive ecotoxicological risk assessment of chemicals is based on a comparison of the hazard of a chemical, expressed as its toxicity or safe levels derived from that (PNEC values), with predicted or measured exposure levels (PEC or MEC). This will provide an assessment of the possible risk predicted or diagnosed for exposed ecosystems. Standards or risk limits for chemicals in the environment generally are based on PNEC values, sometimes lowered by application of a safety or uncertainty factor, depending for instance on the availability of toxicity data (number of data, representation of different species (see also below), endpoints etc.).

Diagnostic approaches are applied to monitor possible effects of chemicals after introduction onto the market, e.g., as post-registration monitoring of pesticides, or to assess the actual risk of contaminated soils. Diagnostic risk assessment, for instance of contaminated soils, relies on a combination of ecological, toxicological and chemical approaches. Basically, toxicity tests (in this case usually called bioassays) are used as diagnosis tools to assess the toxicity of soil samples from the contaminated site. Results of the bioassays are considered together with those of measurements of total or available concentrations of a selected number of chemicals and ecological field observations. The added value of bioassays is that they provide information on the actual risk of bioavailable concentrations of all chemicals present in the contaminated samples; such information cannot be obtained from chemical analyses (see e.g., van Gestel et al. 2001; Loureiro et al. 2005, 2006). Together these approaches result in an assessment of the potential risk of soil contamination, for instance in the TRIAD approach, which combines three lines of evidence: chemistry (total or available concentrations of pollutants), toxicity of the polluted soil and on site ecological observations (Jensen and Mesman 2006).

## Toxicity tests

One key issue in ecotoxicological risk assessment is the selection of test species for generating the required toxicity data. For a proper risk assessment it is crucial that test species are representative of the community or ecosystem to be protected. Criteria for selection of tests and therefore also for organisms to be used in toxicity tests have been summarised by Van Gestel et al. (1997). They include 1. Practical arguments, including issues like feasibility, cost-effectiveness and rapidity of the test, 2. Acceptability of tests, like the need to be reproducible and standardised, and 3. Ecological significance, including sensitivity, biological validity etc.

There is no species that is most sensitive to all pollutants. Which species is most sensitive depends on the mode of action and possibly also other properties of the chemical, and the properties of the organism (e.g., presence of specific targets, physiology, etc.). It is therefore important to always test a number of species, with different life traits, functions, and position within a trophic chain. Such a battery of test species should be (according to Van Gestel et al. 1997):

Representative of the ecosystem to protect, so including organisms having different life-histories, representing different functional groups, different taxonomic groups and different routes of exposure;
Representative of responses relevant for the protection of populations and communities; and
Uniform, so all tests in a battery should be applicable to the same test media and applying the same test conditions.

Once tests have been developed, accepted, and validated, they may be standardised by international organisations like the International Standardization Organization (ISO) or the Organization for Economic Co-operation and Development (OECD). Chemical registration authorities usually only accept results of tests standardised by these organisations. For the soil environment different OECD and ISO standardised tests are available (for an overview see e.g., Van Gestel 2012), including earthworms, enchytraeids, springtails, insect larvae and molluscs as test species. ISO tests are primarily focusing on diagnostic/retrospective risk assessment while OECD tests are required for predictive/prospective risk assessment. Some tests determine short-term or acute exposures, usually with mortality as the endpoint, while others focus on sublethal endpoints like reproduction. Also tests on avoidance behaviour with earthworms (ISO 2008) and springtails (ISO 2011) have been developed as a fast method of assessing another relevant sublethal endpoint.

The ecological relevance of isopods, their typical routes of exposure (soil, food) and life history characteristics, the possibility to determine different endpoints (see below), and the fact that they have already been used for testing for more than 30 years, make them highly suitable test organisms (Drobne 1997; Van Gestel 2012). Unfortunately, so far, no toxicity tests on isopods have reached the level of standardisation, although several methods have been applied in the literature. One reason for the lack of a standardised test with isopods could be their relatively long and more complex life cycle compared to springtails or earthworms. This makes it more difficult, at least for a number of species, to culture them in the lab. For many isopod species it takes several months or more from hatching to reproductive age. Another problem is the rather large variation in test results, which could in part be attributed to the difficulty of obtaining age-synchronised groups of test animals but could also be an intrinsic property of isopods, like the moulting process or their daily excretion process within the hepatopancreas. Both the long life span and the large variation, together with the ability of females to store sperm, make it more difficult to standardise a reproduction toxicity test. Another point which may hamper the standardisation of toxicity tests with isopods may be related to the uncertainty about the main route of exposure (soil vs. food; see below). Nevertheless, there are possibilities of getting around these problems, as will become clear from the following sections.

## Main sources of isopod exposure in soil

In terrestrial systems, several pollutants can reach the soils from diverse sources and can also be found in decaying organic matter. These sources can be considered as point and/or diffuse (non-point) and they will include different forms of contamination: gaseous (atmospheric), solid or liquid hazardous substances that will be mixed with soil.

Industry and commerce are two of the major economic sectors responsible for soil contamination (36 %), either by negligence or by accident (European Environment Agency (EEA); www.eea.europa.eu). This includes atmospheric emissions from production, spilling or burying chemical substances directly in the soil or through runoff from surrounding areas. Such polluted sites are common in Europe and worldwide, and are commonly named as historically-contaminated sites, where metals (37.3 %) and mineral oils (33.7 %) are the main harmful substances to be considered, followed by Polycyclic Aromatic Hydrocarbons (PAHs) (13.3 %), Aromatic Hydrocarbons (BTEX) (6 %), phenols (3.6 %), chlorinated hydrocarbons (2.4 %), and other chemical compounds (3.6 %) (EIONET priority data, EEA). Along with industrial and commercial sources, waste treatment and disposal is another main source of soil contamination (EEA; www.eea.europa.eu). Sewage sludge is often applied to agricultural fields as fertiliser. This provides a considerable input of hazardous substances to soils, some of them considered as emerging pollutants, like those in daily care products, pharmaceuticals, or nanomaterials. In addition, Phthalates (e.g., diethylhexylphthalate (DEHP), dibutylphthalate (DBP)), Octylphenols, Nonylphenols, Linear alkylbenzene sulfonates (LAS), Polychlorinated biphenyls (PCBs), PAHs and metals are amongst the substances most commonly found in sewage sludge that is applied to soils for agricultural purposes. Considering this cocktail of chemicals along with pesticides and fertilisers, agricultural soils are a major sink for contaminants. Pesticides have been introduced by man in ecosystems initially as natural compounds, by using poisonous plants or extracting chemical substances from other natural sources. Later in the 20^th^ century, especially after the 2^nd^ World war, anthropogenically modified or synthesised compounds have been introduced at a large scale, that nowadays represent a wide range of organic chemical groups, including organophosphates, carbamates, triazines, organochlorines, pyrethroids, neonicotinoids, sulfonylurea and biopesticides (EEA; www.eea.europa.eu).

Along with agricultural areas, urban areas have been identified as hotspots for soil contamination (Santorufo et al. 2012), with sources like petrol stations and mechanical workshops where PAHs can be found (Din et al. 2013), traffic and urban roads as sources of metals (e.g., copper, zinc, lead) or changing soil pH (see e.g., Falkengren-Grerup 1986; Løkke et al. 1996; Blake et al. 1999), and agricultural practices in small gardens leading to increasing levels of several contaminants like metals and organic chemical substances. One of these case studies was performed in China where agricultural plastic film was spotted as a possible important source of soil phthalate ester contamination in a suburban area (Wang et al. 2013).

Lately emerging chemicals like nanomaterials (e.g., nanoparticles) are showing a potential risk to aquatic and terrestrial organisms as they are being used for different applications and are expected to appear in the environment, mainly through sewage sludge discharges. Recently also microplastics have been added to this list of pollutants potentially threatening the soil environment (Rillig et al. 2017), although some biodegradable plastics can also be used by isopods as a food source facilitating the degradation of these materials (Wood and Zimmer 2014).

In addition to chemical stress, natural stress can also affect the performance of soil organisms, including isopods. Considering their evolution from water to land, terrestrial isopods have acquired several features to succeed their appearance in terrestrial environments. One limiting habitat property is moisture content, while temperature, salinity, and UV radiation increase are also of importance. These factors can become stressors on their own when tending to extremes but they can also act as joint stressors in addition to chemicals. This joint effect can be caused by the interaction of stressors in exposure media but most interestingly also inside the organisms. It is known that several environmental conditions like temperature fluctuation patterns will influence an organism’s physiology and behaviour, changing therefore the metabolism of chemicals upon exposure or affecting its behaviour, e.g., its aggregation behaviour (Hassall et al. 2012). Donker et al. (1998) showed that zinc was highly toxic to *Porcellioscaber* at higher temperatures mainly due to an increase in isopod metabolism rather than due to the increase of zinc body burdens. UV radiation was also reported to harm isopods (*Porcellionidespruinosus*) by itself, showing that juveniles and pre-adults were more affected than adults, mainly for energy-related parameters (Morgado et al. 2013). Besides temperature, there are only few studies investigating the effects of exposure conditions on the toxicity of chemicals to terrestrial isopods.

## Routes of exposure

In soil, chemicals may be distributed over different compartments, the soil solid phase, pore water, and air. Isopods living on and in the soil may be exposed to all three compartments, with food acting as another compartment from which chemicals may be taken up. Very little data is available on the way isopods are exposed and on what the relative importance of each route of exposure is. Likely, the relative importance of either route of exposure is dependent on the properties of the chemical, including its volatility, water solubility and sorption or soil/water partition constant, and the properties of the soil, like organic matter content, clay content, and pH. As a consequence of these factors, bioavailability and therefore exposure may be quite different for soil and food. For volatile chemicals inhalation may present another route of uptake, which again will be hard to quantify. It therefore remains hard to predict the role of either route of exposure, and this will also require knowledge of several factors related to isopod behaviour (see Løkke and Van Gestel 1998 for some considerations on routes of exposure).

In the toxicity tests described in the literature, generally two routes of exposure have been tested, usually separately: exposure to food only or to soil only (see Table 1 for a summary of literature data on the toxicity of selected chemicals to isopods; Suppl. material 1: Table S1 in the Supporting Information provides a more detailed but not exhaustive overview of toxicity data). In few cases, however, animals were exposed to both treated food and treated soil. In the latter case, it should be noted that it will be very hard to realise the same exposure in terms of bioavailable concentrations in food and soil as properties may also be quite distinct with food generally containing much higher organic matter contents than soil (but with different properties). As a consequence, Fischer et al. (1997) found that dimethoate was more toxic for *Porcellioscaber* when mixed in with soil than with food. This finding was supported by Hornung et al. (1998b) with data for copper and LAS, using the same species, by Sousa et al. (2000) studying the accumulation of lindane in *Porcellionidespruinosus* and by Vink et al. (1995) determining toxicity of benomyl, carbofuran, and diazinon to the latter species.

**Table 1.** Summary of data on the toxicity of chemicals to isopods in different tests with exposures in soil or through food. For each chemical, species and endpoint, the lowest value is reported. For a more complete overview of data, it is referred to the Supporting Information.

Test compound	Species	Soil/food	Time (d)	Criterion	Endpoint	Result (mg/kg dry soil or food)	Reference
2-phenylethyl isothiocyanate	* Porcellio scaber *	food	28	LC50	survival	>1000	Van Ommen Kloeke et al. 2012
		Lufa2.2	28	LC50	survival	65.3	
3-phenylpropionitrile	* Porcellio scaber *	food	28	LC50	survival	>1000	Van Ommen Kloeke et al. 2012
		Lufa2.2	28	LC50	survival	155	
abamectin	* Porcellio scaber *	Lufa 2.2	21	LC50	survival	69	Kolar et al. 2008
		Lufa 2.2	21	NOEC	weight loss	3	Kolar et al. 2010
AgNO_3_	* Porcellionides pruinosus *	food	14	EC50	growth	233	Tourinho et al. 2015
		Lufa 2.2	14	LC50	survival	396	
			14	EC50	consumption	56.7	
			2	EC50	avoidance	13.9	
AgNPs	* Porcellionides pruinosus *	food	14	EC50	growth	>1500	Tourinho et al. 2015
		Lufa 2.2	14	LC50	survival	>455	
			14	EC50	growth	114	
			2	EC50	avoidance	15.8	
benomyl	* Porcellionides pruinosus *	2 Soils	14	LC50	survival	>1000	Jänsch et al. 2005
benzo[a]anthracene	* Oniscus asellus *	food	329	NOEC	growth	3	Van Brummelen et al. 1996a
	* Porcellio scaber *	food	112	NOEC	growth	>9.6	
benzo[a]pyrene	* Oniscus asellus *	food	63	NOEC	growth	10.6	Van Brummelen et al. 1996a
	* Porcellio scaber *	food	63	NOEC	growth	10.6	
bisphenol A	* Porcellio scaber *	sandy soil	112	NOEC	growth	≤10	Lemos et al. 2010
			70	LC50	survival	910	Lemos et al. 2009
carbendazim	* Porcellionides pruinosus *	2 Soils	14	LC50	survival	>1000	Jänsch et al. 2005
Cd	* Armadillidium vulgare *	food	21	NOEC	MT/ HSP70 expression	43.14	Mazzei et al. 2015
	* Oniscus asellus *	food	91	LC50	survival	~1600	Crommentuijn et al. 1994
	* Porcellio scaber *	food	308	LC50	survival	86	Crommentuijn et al. 1995
			70	EC10	growth/biomass	1.35	Abdel-Lateif et al. 1998
			21	LOEC	food selection	20	Zidar et al. 2005
			21	NOEC	moulting/survival	>200	
	* Porcellionides pruinosus *	food	28	EC50	egestion ratio	370	Loureiro et al. 2006
			28	LOEC	assimilation efficiency	19850	
chloranthraniliprole	* Porcellio scaber *	Lufa 2.2	32	LC50	survival	>1000	Lavtizar et al. 2016
			32	NOEC	growth	>1000	
chlorpyrifos	* Porcellionides pruinosus *	Lufa 2.2	14	NOEC	biomass	≥3	Morgado et al. 2016
Cu	* Porcellio scaber *	food	28	EC10	growth	45	Farkas et al. 1996
			28	LC50	survival	1117	
		Lufa 2.2	28	NOEC	growth	500	Hornung et al. 1998a
			28	LC50	survival	3755	
	* Porcellionides pruinosus *	food	28	EC50	consumption ratio	1038	Loureiro et al. 2006
			28	EC50	egestion ratio	483	
			28	LOEC	assimilation efficiency	>10500	
		Lufa 2.2	2	EC50	avoidance behavior	802	Loureiro et al. 2005
dimethoate	* Porcellio scaber *	food	28	LC50	survival	>75	Fischer et al. 1997
			28	NOEC	growth	>75	Hornung et al. 1998
		2 soils	28	EC10	female gravidity	3.8	Fischer et al. 1997
		Lufa 2.2	28	NOEC	growth	10	Hornung et al. 1998a
			28	NOEC	food consumption	10	
	* Porcellio dilatatus *	black silt	2	NOEC	active time	<5	Engenheiro et al. 2005
	* Porcellionides pruinosus *	Lufa 2.2	2	EC50	avoidance behavior	28.7-39.7	Loureiro et al. 2009; Santos et al. 2010
doramectin	* Porcellio scaber *	Lufa 2.2	21	LC50	survival	>300	Kolar et al. 2008
endosulfan	* Porcellio dilatatus *	food	21	NOEC	glycogen / lipids	<0.1	Ribeiro et al. 2001
fluoranthene	* Oniscus asellus *	food	329	NOEC	growth, reproduction	>267	Van Brummelen et al. 1996a
fluorene	* Oniscus asellus *	food	329	NOEC	protein (females)	7	Van Brummelen et al. 1996a
	* Porcellio scaber *		112	NOEC	growth	>219	
glyphosate	* Porcellionides pruinosus *	Lufa2.2	2	EC50	avoidance behavior	39.7	Santos et al. 2010
imidacloprid	* Porcellio scaber *	food	14	NOEC	growth	5	Drobne et al. 2008
		Lufa 2.2	28	LC50	survival	7.6	De Lima e Silva et al. 2017
lambda-cyhalothrin	* Porcellionides pruinosus *	2 Soils	14	LC50	survival	0.5-1.4	Jänsch et al. 2005
			14	EC50	reproduction	0.13-0.4	
lasalocid	* Porcellio scaber *	Lufa 2.2	28	NOEC	growth	202	Žižek and Zidar 2013
			2	NOEC	avoidance behavior	<4.51	
mancozeb	* Porcellionides pruinosus *	Lufa 2.2	14	NOEC	biomass	176	Morgado et al. 2016
Ni	* Porcellionides pruinosus *	Lufa2.2	1-8	NOEC	integrated biomarkers	50	Ferreira et al. 2015
parathion	* Porcellio dilatatus *	food	21	NOEC	AChE	<0.1	Ribeiro et al. 1999
Pb	* Armadillidium vulgare *	food	21	NOEC	MT/ HSP70 expression	478	Mazzei et al. 2015
	* Porcellio scaber *	food	80	NOEC	oxygen consumption	1178	Knigge and Köhler 2000
	* Porcellionides pruinosus *	food	28	EC50	egestion ratio	14050	Loureiro et al. 2006
			28	LOEC	assimilation efficiency	>42070	
			28	LOEC	growth efficiency	>31790	
phenanthrene	* Oniscus asellus *	food	329	NOEC	growth, reproduction	>235	Van Brummelen et al. 1996a
	* Porcellionides pruinosus *	Lufa 2.2	14	LC50	survival	110-143	Tourinho et al. 2015
			14	EC50	biomass	16.6-31.6	
spirodiclofen	* Porcellionides pruinosus *	Lufa2.2	2	EC50	avoidance behavior	0.9	Santos et al. 2010
thiacloprid	* Porcellio scaber *	Lufa 2.2	28	LC50	survival	>32	De Lima e Silva et al. 2017
			28	EC50	consumption	>32	
TiO2 NPs	* Porcellio scaber *	food	3	NOEC	CAT/GST	>3000	Jemec et al. 2008
tributyltin	* Porcellionides pruinosus *	food	14	NOEC	consumption rate	1	Silva et al. 2014
		soil	14	LC50	survival	99.2	
			2	EC50	avoidance behavior	<0.2	
vinclozolin	* Porcellio scaber *	sandy soil	70	LC50	survival	298	Lemos et al. 2009
			35	NOEC	molt delay	10	
Zn	* Porcellio scaber *	food	72	EC50	growth	1916	Van Straalen et al. 2005
			35	NOEC	fecal production	1000	Drobne and Hopkin 1995
	* Porcellionides pruinosus *	food	28	EC50	consumption ratio	11100	Loureiro et al. 2006
			28	EC50	assimilation efficiency	3650	
			28	EC50	egestion ratio	3520	
		4 Soils	14	LC50	survival	1792-2352	Tourinho et al. 2013
			14	EC50	biomass	312-1400	
ZnO non-nano	* Porcellionides pruinosus *	4 Soils	14	LC50	survival	2169-2894	Tourinho et al. 2013
			14	EC50	biomass	119-1951	
ZnO NPs (3–8 nm)	* Porcellionides pruinosus *	4 Soils	14	LC50	survival	1757->3369	Tourinho et al. 2013
			14	EC50	biomass	713-1479	

Food can be an important route of exposure, especially in case of input from the air with freshly fallen decaying leaves containing high levels of pollution. Food exposure may also be more uncertain and more difficult to quantify due to the possibility of isopods avoiding contaminated food. It is known that isopods may be able to survive for very long time without food (Donker 1992), but starvation will of course affect sublethal endpoints like reproduction by affecting energy reserves.

Vijver et al. (2005) demonstrated that surface adsorption of metals is negligible compared to uptake, so dermal uptake seems less relevant than oral uptake unless metals may pass the skin to be internalised rapidly. As mentioned above, soil exposure was shown to be more effective in causing toxicity and bioaccumulation of chemicals in isopods, so it seems that in spite of the low surface sorption, dermal uptake may be an important route of exposure of isopods.

In addition, it should be noted that organisms may affect exposure by their behaviour. Feeding behaviour affects the dietary exposure, while mobility may play a role in determining the degree of contact with soil and therefore may affect soil exposure.

It will be difficult to construct an experiment that completely separates the different exposure routes. This also is not necessary when the focus is not on the mechanisms behind uptake and accumulation but rather on the consequences of exposure in terms of the toxicity. The amount of chemical accumulated instead of the concentration in the environment might provide a suitable measure of exposure and integrates aspects of bioavailability and route of exposure (Escher and Hermens 2004).

It is also not self-evident that the route of exposure will be the same under laboratory and field test conditions. In most standard laboratory tests, the test animal is kept on a relatively thin, homogeneous soil layer and is (if food is provided) forced to feed on a single food item (Hornung et al. 1998a). The only possibility to avoid the polluted food item is to stop feeding, which will only occur when the pollutant makes the food item distasteful or when it is affecting the health of the organism (see e.g., Zidar et al. 2004). In field or mesocosm studies, various food items may be available and test animals may have the ability to escape to less contaminated, deeper layers or to safe micro sites in the heterogeneous soil environment, or simply switch to less toxic food.

## Toxicity testing with isopods: species selection

The first record on relating isopods to terrestrial contamination was a study from Martin et al., in 1976, where the availability and uptake of several metals from woodland litter were recorded and described in the woodlouse *Oniscusasellus*. In 1977, this species was mentioned as a biomonitor of environmental cadmium (Coughtrey et al. 1977) and later, in 1982, *Oniscusasellus* was used by Hopkin and Martin to study the distribution of several metals present in soil. Isopods were mentioned as bioindicators of zinc pollution in England (Hopkin et al. 1986), using species like *Porcellioscaber* and *Oniscusasellus* (Hopkin et al. 1989). *Porcellioscaber* was used as test species in ecotoxicology for the first time in 1978, by Beeby, where the combined uptake of lead and calcium was studied and discussed, continuing later studying the effects of lead assimilation on the brood size of this species (Beeby 1980).

In 1991, *Armadillidiumvulgare* appeared as a test species in a study on the sequestration of copper and zinc in the hepatopancreas, and its relation with previous exposure to lead (Tomita et al. 1991). Vink et al. (1995) discussed the importance of exposure routes and for that used the saprotrophic species *Porcellionidespruinosus*. In 1998, *Porcelliolaevis* appeared as a test species to be used in laboratory experiments where mortality, body mass and behaviour were recorded upon lead exposure (Odendaal and Reinecke 1998). One year later, Ribeiro et al. (1999) described the effects of parathion-ethyl and endosulfan-sulfate on enzyme activities (AChE and LDH) of *Porcelliodilatatus*, also showing that this species was a good candidate for biomarkers in ecotoxicity tests.

*Porcellionidespruinosus* has more recently been proposed as a suitable test species for ecotoxicity testing. The advantage of this species is its somewhat shorter life cycle making it easier to culture, and therefore it is more suitable for performing reproduction toxicity tests (e.g., Jänsch et al. 2005). Although this species seems less abundant in temperate zones, and is more representative of Mediterranean and tropical regions, it can easily be tested at 20 °C, which is the standard temperature for most soil toxicity tests.

Based on the above, it remains difficult to recommend one or the other species for toxicity isopod testing. A toxicity test with isopods may therefore use different species, but its duration and design may differ depending on the species chosen.

## Toxicity testing with isopods: exposure set-up and endpoints

**Figures 1–2.**
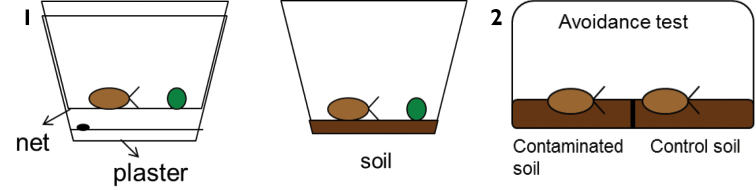
**1** Design of feeding inhibition tests with isopods, applying exposure through food (left) or to contaminated soil with contaminated or uncontaminated food (right). In the test with contaminated food only, the animals are kept on a net or gauze allowing also for collecting faeces produced; this will enable estimating food assimilation efficiency. By offering the animals pre-weighed disks or pieces of leaf, food consumption can easily be determined. **2** Design of an avoidance test with isopods. The test uses containers with two compartments. One compartment is filled with contaminated soil, the other one with clean soil. After two days of exposure, the position of the animals in the container is checked. By testing a range of concentrations, including a control (clean soil in both compartments), a dose-response relationship for avoidance may be obtained. The test may also be used to assess avoidance responses to field-contaminated soils, but in that case it might be more difficult to find a proper control soil. Drawing made by Paula Tourinho.

Although no standard test guidelines are available for assessing chemical toxicity to isopods, they are used as test organisms, applying different routes of exposure (food, soil), different test durations, and different endpoints. Table 2 provides an overview of different isopod toxicity tests and the endpoints determined as described in the literature. Figures 1–2 show some typical experimental setups employed for toxicity testing with isopods.

Toxicity tests with isopods in soil use either artificial soil, prepared following the methods described by OECD (1984), or natural soils. Since the work of Hornung et al. (1998a), the use of the German standard soil Lufa 2.2 seems most preferred. This soil has a fairly constant composition, with properties appearing less variable than those of artificial soils prepared by different laboratories (see e.g., Bielska et al. 2012). Generally chemicals are mixed in with the soil at different concentrations to assess toxicity. Food exposure uses different matrices, ranging from intact tree leaves (or pieces of leaf) to finely ground leaf material or pellets prepared from tree leaves, sometimes mixed with some other materials. In case intact pieces of leaf are used, they are either soaked, smeared or sprayed with solutions containing different concentrations of the test chemical to achieve a range of exposure. Ground leaves of pelleted food may be spiked by mixing in the test chemicals at different concentrations. In all cases, both soil and food, a control without test chemical is included. In case the chemical has low water solubility and spiking the chemical required a solvent, a solvent control is included as well. Another methodology has been also applied by mixing chemical powder directly in soil and afterwards adjusting its moisture content, which has proved to be a good and reliable technique (Loureiro et al. 2005).

Toxicity tests with isopods have been carried out with several endpoints (Tables 1, 2). Along with the systems biology approach, exposures have been carried out using endpoints at the individual, organ, tissue, and cell or even at the molecular level. Tests focusing on whole organism responses (as shown in Table 2) usually range from 7 to 28 days and evaluate parameters like growth, feeding activity, moulting, mortality or behaviour and > 28 days when reproduction is the endpoint (e.g., Hornung et al. 1998a; Drobne and Hopkin 1995; Drobne and Štrus 1996; Loureiro et al. 2005). These parameters are considered ecologically relevant and usually related to the specific ecological properties of isopods or/and their role in soil function. As examples, feeding inhibition tests are closely related with the isopods’ function as detritivores, and reduced feeding rate as a consequence of either avoidance or intoxication by ingestion of contaminated food is related to the soil habitat function. Feeding inhibition tests were first developed using contaminated food as exposure route. This could be somehow difficult to transpose to real contamination scenarios and therefore new strategies for using this ecologically relevant parameter were developed. New studies on the effects of soil contamination on the feeding activity of isopods have been carried out, along with effects on their biomass (Tourinho et al. 2013).

**Table 2.** Overview of isopod toxicity and bioaccumulation test methods described in the literature. References are just given as an example; in many cases several papers are available describing a more or less similar method of testing. For an overview of toxicity data generated using these methods, see Table 1 and also Table S1 in the supporting information.

Toxicity test	Species	Age	Exposure time (days)	Route of exposure	Endpoints	Test validity criteria	References
acute toxicity	* Porcellionides pruinosus *	adult	14	soil	survival		Tourinho et al. 2013
growth toxicity	* Porcellio scaber *	juvenile	28	soil (artificial and natural)	survival, biomass change	control mortality <20%	Hornung et al. 1998a
				food pellets, leaf litter	survival, biomass change	control mortality <20%	
reproduction toxicity	* Porcellio scaber *	adult	up to 70	soil (artificial and natural)	survival, oosorption, gravid females, offspring	control mortality <20%	Hornung et al. 1998a
				food pellets, leaf litter	survival, oosorption, gravid females, offspring	control mortality <20%	
	* Porcellio dilatatus *	adult	54	food (lettuce incorporated in gelatine)	survival, time to pregnancy, pregnancy duration, abortions, juveniles		Calhôa et al. 2012
feeding inhibition	* Porcellio scaber *	adult	21	food (pellets)	food consumption rate, chemical assimilation, growth, moulting and survival		Zidar et al. 2005
	* Porcellionides pruinosus *	pre-adult	14	soil	consumption rate, assimilation rate, biomass change		Tourinho et al. 2013; Silva et al. 2014
				food (leaf litter)	consumption rate, assimilation rate, excretion rate, biomass change		Loureiro et al. 2006
feeding inhibition	* Porcellio scaber * * Oniscus asellus *	adult	35	food (leaf litter)	feeding rate, excretion rate, assimilation efficiency, accumulation, chemical ingestion		Drobne and Hopkin 1995
	* Porcellio scaber *	adult	28	food (leaf litter or pellets)	body mass gain, food consumption, gravid females, juveniles		Farkas et al. 1996
			84	food (leaf litter or pellets)	survival		
avoidance behaviour	* Porcellionides pruinosus *	adult	2	soil	% avoidance, habitat function	no avoidance in control vs control	Loureiro et al. 2005
foraging behaviour	* Porcellio scaber *	adult	2	food	preference (video tracking)		Zidar et al. 2005
bioaccumulation	* Porcellionides pruinosus *	pre-adult	40 (21 uptake; 19 elimination)	soil	bioaccumulation, kinetics		Sousa et al. 2000
		pre-adult	41 (21 uptake; 20 elimination)	food (leaf litter)	bioaccumulation, kinetics		
bioaccumulation	* Porcellio scaber *	adult	32 (16 uptake; 16 elimination)	Leaf powder	bioaccumulation kinetics		Kampe and Schlechtriem 2016

Avoidance response behaviour to contaminated food or soil is also the fastest endpoint, with test durations of no more than two days. No standard test guideline for avoidance tests with isopods is available, but such tests have been done by e.g., Loureiro et al. (2005) and Zidar et al. (2005) using *Porcellionidespruinosus* and *Porcellioscaber*, respectively. The latter test did not only focus on avoidance but also included foraging behaviour as an endpoint. Avoidance tests may be as sensitive as reproduction for some chemicals, while for others it is at least as sensitive as survival. Behavioural response studies with isopods have been developed due to organisms’ ability to detect chemicals, by using their chemoreceptors located on the second antenna, as a major advantage for establishing avoidance behaviour tests (Loureiro et al. 2005). The methodology proposed by Loureiro et al. (2005) was adapted from the earthworm avoidance behaviour test (ISO 2008). It is easy to perform and suitable to evaluate laboratory soils spiked with organic chemicals, metals, single and as binary mixtures (Loureiro et al. 2009), but also with natural contaminated soils from an abandoned mine (Loureiro et al. 2005).

Another interesting endpoint that is directly related to decomposition is the microbiome in the isopod gut (Drobne et al. 2002). This may also provide information on how colonisation and further decomposition of isopod faeces in the environment is influenced by the presence of chemicals.

In addition, mortality, growth, and reproduction are more related to the population level and bridge the gap to higher levels of biological organisation. These studies are more difficult to perform, as mentioned above, due to the life-span of isopods and their moulting behaviour. Reproduction tests with isopod have been performed in a 48-week exposure test via food contaminated with PAHs using *Oniscusasellus* (Van Brummelen et al. 1996a), and by Lemos et al. (2010) using *Porcellioscaber* exposed to bisphenol A and vinclozolin.

Looking at lower organisational levels, biomarkers are defined as any measurable biochemical, cellular, histological, physiological or behavioural change that can provide evidence of exposure and/or effects from one to more contaminants (Van Gestel and Van Brummelen 1996). Different kinds of biomarkers have been studied in isopods and some were successfully implemented in field studies. The isopods’ hepatopancreas has been used as a key organ to evaluate deleterious effects due to chemical bioaccumulation. The hepatopancreas is a storage organ where detoxified, sequestered chemicals (metals) can be stored but it also plays an important role in the animal’s metabolism. The hepatopancreas contains two cell types, the Big cells (B cells) and the Small (S cells), that differ in their excretion behaviour. The S cells are known to accumulate large amounts of metals, mainly related to metal storage, and B cells are renewed frequently, therefore playing the main role in excretion (Hopkin and Martin 1982). The histopathological changes of isopods’ digestive glands have also been characterised and studied (Drobne and Strus 1996; Odendaal and Reinecke 2003, 2004; Lapanje et al. 2008; Lešer et al. 2008). The neonicotinoid insecticide imidacloprid showed to affect the digestive gland epithelial thickness in *Porcellioscaber* (Drobne et al. 2008). In addition, and for the species *Porcellionidespruinosus* the mean percentage of cellular area (thickness) of digestive gland epithelia was also significantly lower in animals from the smelting work compared to animals from unpolluted sites. Later studies revealed that epithelial thickness can be significantly affected by starvation (Lešer et al. 2008).

In order to study endocrine disruptor effects, Lemos et al. (2009) studied moulting behaviour and total ecdysteroid (20E) concentration of *Porcellioscaber* upon exposure to bisphenol A and vinclozolin. At the molecular or enzymatic level, isopods have also been used to evaluate the response to oxidative stress and neurologic effects. Several biomarker methodologies have been adapted from other organisms’ protocols to be used in isopods. Stress proteins (e.g., heat-shock proteins; HSPs) were studied in *Oniscusasellus* as a molecular marker of multiple metal exposure, while cholinesterase activity was measured in *Porcelliodilatatus* upon exposure to dimethoate and related with locomotor activity (Engenheiro et al. 2005). Other biomarkers have also been used to evaluate chemical exposure, such as lysosomal membrane stability (Nolde et al. 2006), glutathione S-transferase (GST) or catalase (Jemec et al. 2008), or energy reserve contents, by studying the effects of chemicals on the lipid, protein and glycogen contents (Donker 1992). To improve biomarker methodologies in isopods and also to use basal levels as foundations for isopod exposure or just for isopod health status in cultures, Ferreira et al. (2010) characterised the basal levels of several biomarkers and energy reserves in *Porcellionidespruinosus*.

These biomarkers have been used to detect effects of individual chemicals but also to unravel modes of actions of chemicals and explain their effects when present in the environment as mixtures. In the study of Santos et al. (2010a), molluscicide baits induced extreme effects to the isopod *Porcellionidespruinosus*. In this study glutathione S-transferase (GST), acetylcholinesterase (AChE) and catalase (CAT) were analysed upon exposure to single chemicals and to binary mixtures. Although the carbamate methiocarb significantly inhibited AChE activity, no oxidative stress was detected (by using CAT and GST levels). On the other hand, metaldehyde showed a completely different mode of action with no effects on AChE, but inducing a decrease in GST activity as well as a general increase in CAT activity. The combined exposure to the two molluscicides resulted in a general decrease in AChE and CAT activity, but no visible effects were observed in terms of GST activity.

HSPs, initially discovered in salivary glands of *Drosophila* exposed to heat (Ritossa 1962), were intensively investigated in isopods in the 1990s (Köhler et al. 1992, 2000; Köhler and Eckwert 1997; Eckwert and Köhler 1997; Knigge and Köhler 2000; Arts et al. 2004; Weeks et al. 2004). HSPs in *Porcellioscaber* and *Oniscusasellus* from metal-polluted and unpolluted sites were analysed and compared with semi-field (microcosm) and laboratory studies. In field populations a high inter-site variability of the hsp70 level was detected. The stress response level was positively correlated with metal solubility, C/N ratio and negatively correlated with soil pH and with site-specific pollution history-adaptation (Köhler et al. 2000; Arts et al. 2004; Weeks et al. 2004). Potential adaptation in HSP responses was demonstrated previously in a laboratory study where *Oniscusasellus* from unpolluted and polluted sites were exposed to metals. In non-adapted populations the level of stress proteins increased after exposure to metals, while in metal adapted populations the induction of stress proteins was less prominent or even decreased (Eckwert and Köhler 1997). Some interspecies variation in HSP level was reported as well (Arts et al. 2004; Weeks et al. 2004). In *Porcellioscaber* collected from the vicinity of smelter, the HSP level was comparable to control animals while in *Oniscusasellus* from the same location it was much higher compared to *Porcellioscaber* and control animals (Arts et al. 2004). Arts et al. (2004) concluded that hsp70 level is a suitable biomarker of effect in non-adapted and adapted populations but of exposure only in non-adapted individuals.

In addition to HSPs, several other biomarkers were studied *in situ* in relation to predominantly metal(s) contamination. Metal storage granules and energy reserves were investigated in *Oniscusasellus* and *Porcellioscaber* in relation to distance to the smelter at Avonmount, UK (Schill and Köhler 2004). *Porcellioscaber* and *Oniscusasellus* showed different response to metal pollution, which was in accordance to what was achieved by Arts et al. (2004). In *Oniscusasellus* the number and size of metal granules increased with decreasing distance to the smelter while the amount of lipids and glycogen decreased. None of this was found in *Porcellioscaber*. Nolde et al. (2006) and Lapanje et al. (2008) investigated lysosomal membrane stability (LMS) of digestive gland cells in *Porcellioscaber* as a biomarker of effect. LMS in animals from highly mercury polluted environments was less affected compared to animals from moderately or unpolluted environments when exposed to mercury. Besides LMS, gut bacterial structure was less affected as well (Lapanje et al. 2008).

## Bioaccumulation of chemicals in isopods

**Figure 3.**
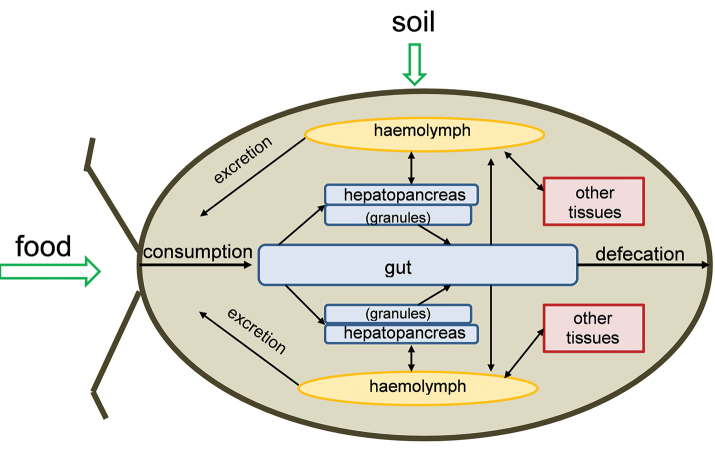
Schematic overview of the routes of uptake and internal processing of chemical pollutants in isopods. Adapted from Donker et al. (1996).

Considering the extremely high metal concentrations found in animals from contaminated areas (see e.g., Hopkin 1989), metal bioaccumulation in isopods has been studied to a great extent. These studies focused on several elements. Initial studies were mainly restricted to assessing whole body metal concentrations in isopods exposed for a fixed time to contaminated soil or food. Later studies also included metal uptake and elimination kinetics, sometimes also in relation to the internal distribution of metals inside the isopod’s body (see e.g., Figure 3). Finally, several studies focused more in detail on the mechanisms of metal sequestration in hepatopancreas tissues.

Isopods have been shown to have a tremendous capacity of storing metals in the hepatopancreas. As mentioned above, the hepatopancreas plays an important role in metal sequestration. Due to the high storage capacity of the hepatopancreas, metal uptake kinetics tends to be fairly slow in isopods. Basically, this means that it takes a long time to reach equilibrium, as was shown for cadmium (Vijver et al. 2006). This high capability of sequestering metals is strongly related to storage of the metal in inert fractions, as was shown for zinc and cadmium (Vijver et al. 2006). As a consequence, the lethal body concentration for cadmium is very high in isopods (*Porcellioscaber*, *Oniscusasellus*) compared to other soil arthropods (Crommentuijn et al. 1994). This also explains why metal concentrations in isopods from contaminated sites can reach very high levels without causing the population to go extinct. But it also means that static tests with fixed exposure times will never be able to provide insight into body concentrations to be expected in field-exposed animals. At best, such tests may provide Bioaccumulation Factors (BAFs), relating body concentrations to soil or litter concentrations. Since kinetics is slow, BAFs for metals in isopods will be time-dependent. For that reason, it is preferred to estimate BAFs on the basis of uptake and elimination kinetics parameters derived from toxicokinetics tests rather than from static tests. In addition, it is well known that BAFs for metals are also concentration-dependent (McGeer et al. 2003).

Metal speciation in food may have an effect on metal uptake in isopods, as was for instance shown by Calhôa et al. (2006) and Monteiro et al. (2008). These studies demonstrate that Cd speciation and subcellular distribution in plants influence the assimilation efficiency of Cd in the isopod *Porcelliodilatatus*. Only few studies have addressed the potential for food chain transfer of the inert metal fractions in the hepatopancreas of isopods. Bioaccumulation of organic compounds in isopods has been determined in several studies, but only few studies were performed to assess uptake and elimination kinetics of organic compounds in isopods (e.g., Van Brummelen and Van Straalen 1996; Sousa et al. 2000; Loureiro et al. 2002; Santos et al. 2003; Stroomberg et al. 2004; Kampe and Schlechtriem 2016). From the available literature, it is clear that isopods do show high uptake of metals like Cd, but less of Pb and uptake of organic chemicals may be quite variable. The studies of Van Brummelen and Van Straalen (1996) and Stroomberg et al. (2004) demonstrated that isopods (*Porcellioscaber*) have a high capacity of biotransforming PAHs. As a result these compounds are rapidly eliminated from the isopod’s body and PAH concentrations tend to be fairly low in animals from polluted sites when compared to other soil invertebrates like earthworms (Van Brummelen et al. 1996b).

Test methods for determining the uptake and elimination kinetics of organic chemicals in isopods are summarised in Table 2. The methods use either soil or food exposure, and include an uptake phase during which the animals are exposed to contaminated media followed by an elimination phase on clean media. In addition to the species mentioned in Table 2, *Porcellionidespruinosus*, also other isopod species could be used. Bioaccumulation kinetics approaches may also be used to assess the bioavailability of contaminants in soils. As proposed by Van Straalen et al. (2005) the uptake rates (or initial slope of the uptake curve) determined in uptake and elimination tests may provide an indication of metal bioavailability. Animals can be exposed to soil from the field under controlled laboratory conditions, but such an approach may also be applied to freshly spiked soils. A similar approach was used by Udovič et al. (2013) to assess the remediation efficiency. The disadvantage of such approach is that it neglects the impact of natural environmental conditions. This disadvantage could however, also be seen as an advantage: the bioassay provides an integrated assessment of the situation at the time of sampling, but it could be much more expensive, time- and labour-consuming than just performing chemical measurements.

## The use of isopods in microcosm/mesocosm-based toxicity tests

Single species toxicity tests have several shortcomings (adapted from Van Leeuwen and Vermeire 2007) among others:

they are performed under stable and controlled laboratory conditions that are not similar to natural conditions;
interactions between different species and interaction with natural stressors are not taken into account;
the distribution and degradation of chemicals is ignored;
usually genetically homogeneous laboratory raised animals are used.

In contrast to single species toxicity tests microcosms, mesocosms or macrocosms are small, medium, or large multispecies systems that simulate natural situations to a certain degree (Walker et al. 2012). They are more ecologically relevant and compared to full-scale field tests less complex and less expensive. They can be performed in a controlled laboratory environment or under the field conditions. Microcosms with isopods were designed from small containers filled with soil (Eckwert and Köhler 1997, Köhler et al. 2000; Arts et al. 2004; Engenheiro et al. 2005) or sand (Van Wensem 1989) with partly decayed leaves on the surface to complex systems with several plant and animal species (Gunderson et al. 1997; Foerster et al. 2006; Domene et al. 2010; Santos et al. 2011). Uncontaminated and artificially contaminated soils (Santos et al. 2011) or field-contaminated soils (Köhler and Eckwert 1997; Köhler et al. 2000; Arts et al. 2004) were used. Microcosms were exposed to the field conditions (Eckwert and Köhler 1997, Köhler et al. 2000; Arts et al. 2004), to greenhouse semi-field conditions (Gunderson et al. 1997; Engenheiro et al. 2005) or performed in the laboratory (Foerster et al. 2006; Santos et al. 2011). Chemicals were applied by spraying (Santos et al. 2011) or introduced into the soil (Domene et al. 2010). Besides mortality, growth, and reproduction (Gunderson et al. 1997; Foerster et al. 2006), carbon dioxide production (Van Wensem 1989), locomotor behaviour (Engenheiro et al. 2005) and diverse molecular biomarkers (Köhler and Eckwert 1997; Köhler et al. 2000; Arts et al. 2004; Engenheiro et al. 2005; Santos et al. 2011) were measured.

The main disadvantage of such multispecies tests is that the more they imitate a natural environment the more difficult they are to replicate and to standardise. Microcosm tests are higher tier tests, usually designed to test a specific hypothesis, and not to be used routinely.

## Isopods in field studies and biomonitoring

Field studies may take both a predictive and a diagnostic approach. In case of a predictive approach, field studies are just the next step after micro- or mesocosm-based toxicity tests described above. Such tests have rarely been done with isopods. Diagnostic field studies are mainly performed within the framework of monitoring, in order to assess the occurrence of effects at (contaminated) field sites. This section will mainly focus on the latter approaches.

Field (*in situ*) studies on the effects of pollutants on biota bridge the gap between laboratory-conducted toxicity studies and abiotic measuring of pollution. An increased concentration of a pollutant in the environment does not necessarily mean disruptive effects to biota. To cause toxic effects, a chemical needs to be sensed or taken up by the organism; therefore, bioavailability is crucial for toxicity. Moreover, in the field organisms simultaneously respond to a variety of anthropogenic and also natural stressors with antagonistic and synergistic actions among them. Therefore, biological monitoring is important to measure the disruptive effects of pollutants to biota. There are four main approaches to biological monitoring of pollution (adapted from Hopkin 1993; see also Walker et al. 2012):

Monitoring the changes in community structure; absence or presence of a particular species indicates particular pollution.
Measuring the concentration of pollutants in a tolerant indicator species; body concentrations indicate bioavailability of pollutants and also indicate the toxicity level to other more sensitive related organisms.
Measuring the effects of pollutants on organisms; physiological, biochemical, cellular and other markers at the organism level can be used as a screening tool in monitoring.
The detection of genetically different populations of species that have evolved resistance in response to a pollutant.

In 1975 a marine monitoring scheme ‘The mussel watch’ was proposed to follow the level of marine contamination with metals, artificial radionuclides, petroleum and chlorinated hydrocarbons (Goldberg 1975). This has led to a considerable effort to find a terrestrial invertebrate group equivalent to mussels to monitor contamination and bioavailability of metals on land. Attributes of mussels that also terrestrial invertebrates had to fulfil were: common and widespread, large populations, resistant to pollutants, bioaccumulation, and long half-lives of pollutants once accumulated in the body. Isopods, already known at that time by their remarkable ability to accumulate Cu (Wieser 1961, 1968; Wieser and Makart 1961), were soon suggested as a useful tool for monitoring available Cu (Wieser et al. 1976), as well as Cd and Pb (Martin et al. 1976) in contaminated ecosystems. This suggestion was supported by further work of Wieser et al. (1977) and Coughtrey et al. (1977) on Cu and Cd, respectively. The authors analysed soil and litter metal concentrations in several polluted regions (around smelters and mines) in Austria and England and compared them with concentrations in isopods from the same locations. Mainly three species of isopods were examined: *Tracheoniscusratkei*, *Oniscusasellus* and *Porcellioscaber*. Total body concentrations of metals and concentrations in different body parts (hepatopancreas, gut, ovaries, and exoskeleton) were analysed. The main findings were:

concentrations of Cu and Cd in isopods increase with increasing metal concentrations in litter (Martin et al. 1976; Wieser et al. 1976, 1977; Coughtrey et al. 1977);
the Bioaccumulation Factor isopods/litter (BAF) for Cu (Wieser et al. 1977) and Cd (Martin et al. 1976) is around 6, the highest among terrestrial invertebrates;
mean Cu content in isopods is highly correlated with Cu concentration in litter but less with Cu concentration in soil (Wieser et al. 1977);
Cu and Cd content in isopods is related to body weight (Wieser et al. 1976, 1977; Coughtrey et al. 1977); and
Cu content in isopods fluctuates with season and it is temperature-dependent (Wieser et al. 1977).

Almost ten years later, results from a large field study were published (Hopkin et al. 1986). Concentrations of Zn, Cd, Pb and Cu in litter from 89 sites around smelting works in the Avonmouth area, south-west England were mapped together with metal concentrations in *Porcellioscaber* (hepatopancreas and whole animal concentrations) (Hopkin et al. 1986). In the next study by Hopkin et al. (1993) Zn, Cd, Pb, and Cu concentrations in *Porcellioscaber* were compared with concentrations in *Oniscusasellus* from the same sites and correlated with soil concentrations. Main findings of these studies were:

concentrations in isopods also correlate with soil or litter concentrations of Zn and Pb, like Cd and Cu (Hopkin et al. 1986, 1993);
Zn, Pb, Cd, and Cu concentrations in the hepatopancreas correlate with whole animal concentrations at all sites (Hopkin et al. 1986), thus analyses of separate body parts are not necessary;
BAFs for Zn and Pb are much lower compared to Cu and Cd (Hopkin et al. 1986);
BAFs related to litter are much higher compared to soil and vary greatly between locations; and
5. correlation between Porcellioscaber and Oniscusasellus is closer than between isopods and soil, therefore concentrations in Oniscusasellus and probably also in other invertebrate groups can be predicted from those in Porcellioscaber (Hopkin et al. 1993).

Isopods were also studied in urban areas as bioindicators for Zn (Hopkin et al. 1989) as well as Pb and Cd pollution (Dallinger et al. 1992). *Porcellioscaber* and *Oniscusasellus* were sampled from 63 sites in Reading, England (Hopkin et al. 1989) and 356 points over the city of Innsbruck, Austria (Dallinger et al. 1992). As leaves were not present in most of the urban sites, soil was sampled at the same locations. The main findings were:

Zn concentrations in Porcellioscaber were about two times higher than those in Oniscusasellus at each site (Hopkin et al. 1989);
sources of Zn pollution can be identified according to soil or isopod samples on a small scale (0.5 km apart) but not on a large scale (5 km apart) (Hopkin et al. 1989);
Pb concentrations in isopods correlate with traffic density in individual districts of the city (Dallinger et al. 1992).

Field studies where isopods were used in monitoring of contamination with organic chemicals are very rare compared to metal contamination. Some laboratory studies showed that isopods may also accumulate organic chemicals, like veterinary pharmaceuticals (Kolar et al. 2010) or rodenticides (Brooke et al. 2013). The antiparasitic abamectin accumulated in *Porcellioscaber* in a dose-dependent manner (Kolar et al. 2010) but no data about its retention time were provided. The anticoagulant rodenticide brodifacoum that accumulates in isopods (different species) was measurable even after 45 days after exposure (Brooke et al. 2013). Bioaccumulation of PAHs was also shown (Van Brummelen et al. 1996b), but concentrations were relatively low compared to e.g., earthworms, which might be attributed to the high capability of isopods to biotransform and eliminate these compounds (Van Brummelen and Van Straalen 1996; Stroomberg et al. 2004).

All these studies showed that isopods, particularly *Porcellioscaber*, have favourable attributes to become a leading organism in terrestrial biomonitoring (see e.g., Paoletti and Hassall 1999), especially of metal contamination. Nonetheless, they never reached the status of *Mytilusedulis* in marine biomonitoring, just as no other terrestrial invertebrate group has. There are several reasons for that. All the studies mentioned above showed a certain discrepancy between metal concentrations in isopods and in soil or litter from different locations with comparable metal contamination. As the digestive system is the main route for metal intake in terrestrial isopods (reviewed by Hopkin 1989), their internal metal concentration is actually a measure of metal availability in their food source and not in the soil. Metal concentrations in isopods were in fact more significantly correlated with metal concentrations in litter than in soil. In the field, the isopod diet consists mainly of leaf litter, but the exact food source is often hard to define and in some urban locations also hard to collect. Besides, animals are mobile and their resting locations are not necessarily their feeding locations. To make things even more complex, isopods also discriminate food sources due to different plant species, plant defences (e.g., tannins) and state of microbial decay (Hassall and Rushton 1984; Gunnarsson 1987; Szlávecz and Maiorana 1990) and can probably also differentiate certain contaminants (Loureiro et al. 2005; Zidar et al. 2012; Žižek and Zidar 2013), including metals (Zidar et al. 2005).

The previously mentioned studies also demonstrated a strong correlation between body metal concentrations and isopod body mass. It was therefore suggested to compare animals from the same weight class (Wieser et al. 1977) or to use the slope of regression lines (weight – metal concentration) rather than mean metal concentration for comparison between locations, as population-size structure from different locations may vary (Coughtrey et al. 1977). But size and weight are no reliable markers for age, which indicates time of exposure. The overlap of age classes could occur when the weight of younger but better fed isopods exceeds the weight of individuals born in the previous year (Hopkin 1989). Overlap of age classes might also explain the seasonal fluctuation of copper content in isopods with the maximum in winter and minimum in summer (Wieser et al. 1977), when this year’s generation of isopods already starts to reproduce. Witzel (1998) reported that accumulation of Pb and Cd in *Porcellioscaber* shows two different phases. Up to the age of 2–3 months assimilation exceeds the rate of growth and leads to rapidly increasing concentrations. After 3 months (at a mass of around 5 mg fresh weight) the rate of accumulation is proportional to the rate of growth and the heavy metal concentrations remain on a stabilised level. Besides, size of isopods varies between polluted and unpolluted sites. Donker et al. (1993) reported that females from *Porcellioscaber* populations near a Pb mine and a Zn smelter reproduced at lower weight. This might indicate slower growth or earlier maturity compared to females from clean environments. A significant difference in the mean and maximum size of *Porcellioscaber* has also been reported among sites in the Avonmouth area in England (Jones and Hopkin 1998). At the most polluted sites populations with the smallest maximum size were found, which might be related to higher energy expenditure for detoxification but also to a genetic differentiation of populations in polluted environments.

In the terrestrial environment metal contamination on one side and isopod distribution on the other are influenced more prominently by local environmental conditions compared to marine ecosystems. Amount and distribution of rainfall during the year, wind directions and relief of the landscape together with soil chemistry and vegetation influence the deposition, retention, and availability of metals to biomonitoring organisms on one side and appropriate conditions for their living on the other. All this makes ‘a global woodlouse watch scheme’ (Hopkin et al. 1993) even more difficult. Nevertheless, attempts to use isopods as indicators of metal pollution are still being made, as for instance shown by a study on the use of isopods as an indicator of mercury pollution (Pedrini-Martha et al. 2012; Longo et al. 2013).

Faber et al. (2013) proposed a conceptual approach for implementing ecosystem services in monitoring frameworks. They showed that isopods can also be included in monitoring soil quality and in that way contribute to the assessment of possible effects of land use and other human activities on ecosystem services.

## Conclusions

Isopods are important organisms in terrestrial ecosystems. For that reason they should be considered as test organisms in soil ecotoxicology. A standardised test with isopods could be a relevant and important addition to the existing battery of toxicity tests with soil invertebrates. The difficulties in culturing and testing could be overcome by selecting species with shorter life cycles, like *Porcellionidespruinosus*, and by putting more effort in optimising culture conditions for species like *Porcellioscaber*. This may also help developing standardised toxicity tests that include more relevant endpoints like reproduction, in addition to growth and feeding activity. Little insight exists in the difference in sensitivity of isopod species to different chemicals. The harmonisation between exposure time, the existence of validation criteria based on basal levels for optimum exposure (considering temperature and time) and common endpoints could be a step forward for the accuracy improvement and comparison between studies. Isopods are also relevant and useful organisms for use in field monitoring approaches, for instance to assess the bioavailability of metals, possible (post-registration) effects of pesticide use, exposure to (mixtures of) chemicals and in biological soil quality networks aimed at protecting ecosystem services.

## Supplementary materials

Supplementary material 1 Supporing information Data type: references Explanation note: Table S1: Overview of literature data on the toxicity of selected chemicals to isopods exposed through food or soil. This is an open access article distributed under the terms of the Creative Commons Attribution License (CC BY 4.0), which permits unrestricted use, distribution, and reproduction in any medium, provided the original author and source are credited.van Gestel CAM, Loureiro S, Zidar P
