# Supplementary material for: Terrestrial isopods as model organisms in soil ecotoxicology: a review
Source: Zookeys. 2018 Dec 3;(801):127–62. doi: 10.3897/zookeys.801.21970 (PMC6288250; doi:10.3897/zookeys.801.21970)
Supplement: Supplementary material 1 — Supporing information [file zookeys-801-127-s001.docx]

Supporting Information to

**Terrestrial isopods as model organisms in soil ecotoxicology: a review**

Cornelis A.M. van Gestel^1*^, Susana Loureiro^2^ and Primoz Zidar^3^

^1^ Department of Ecological Science, Faculty of Science, Vrije Universiteit, De Boelelaan 1085, 1081 HV Amsterdam, The Netherlands

^2^ University of Aveiro, Department of Biology and the Centre for Environmental and Marine Studies, Campus Universitário de Santiago, 3810-193, Aveiro, Portugal

^3^ Department of Biology, Biotechnical Faculty, University of Ljubljana, Večna pot 111, SI-1000 Ljubljana, Slovenia

Corresponding author: Cornelis A.M. van Gestel ([kees.van.gestel@vu.nl](mailto:kees.van.gestel@vu.nl))

**Table S1:** Overview of literature data on the toxicity of selected chemicals to isopods exposed through food or soil. The table includes data on exposure through soil and food, using different isopod species, different exposure times, different endpoints etc.

See attached Excel sheet for the table.

**References to the Table S1:**

Abdel-Lateif HM, Donker MH, Van Straalen NM (1998) Interaction between temperature and cadmium toxicity in the isopod *Porcellio scaber*. Functional Ecology 12: 521-527. DOI: 10.1046/j.1365-2435.1998.00227.x

Calhôa CF, Soares AMVM, Loureiro S (2012) Effects on survival and reproduction of *Porcellio dilatatus* exposed to different Cd species. Ecotoxicology 21: 48-55. DOI 10.1007/s10646-011-0762-6

Crommentuijn T, Doodeman CJAM, Doornekamp A, van der Pol JJC, Bedaux JJM, van Gestel CAM (1994) Lethal body concentrations and accumulation patterns determine time-dependent toxicity of cadmium in soil arthropods. Environmental Toxicology and Chemistry 13: 1781-1789. DOI 10.1897/1552-8618(1994)13[1781:LBCAAP]2.0.CO;2

Crommentuijn T, Doodeman C, Van der Pol JJC, Doornekamp A, Rademaker MCJ, Van Gestel CAM (1995) Sublethal sensitivity index as an ecotoxicity parameter measuring energy allocation under toxicant stress – application to cadmium in soil arthropods. Ecotoxicology and Environmental Safety 31:192-200. DOI: 10.1006/eesa.1995.1062

De Lima e Silva C, Brennan N, Brouwer JM, Commandeur D, Verweij RA, Van Gestel CAM (2017) Comparative toxicity of imidacloprid and thiacloprid to different species of soil invertebrates. Ecotoxicology 26: 555-564. DOI: 10.1007/s10646-017-1790-7

Drobne D, Hopkin SP (1995) The toxicity of zinc to terrestrial isopods in a “standard” laboratory test. Ecotoxicology and Environmental Safety 31: 1-6. DOI 10.1006/eesa.1995.1037

Drobne D, Blazic M, van Gestel CAM, Leser V, Zidar P, Jemec A, Trebse P (2008) Toxicity of imidacloprid to the terrestrial isopod *Porcellio scaber* (Isopoda, Crustacea). Chemosphere 71: 1326–1334. DOI 10.1016/j.chemosphere.2007.11.042

Engenheiro EL, Hankard PK, Sousa JP, Lemos MF, Weeks JM, Soares AMVM (2005) Influence of dimethoate on acetylcholinesterase activity and locomotor function in terrestrial isopods. Environmental Toxicology and Chemistry 24: 603-609. DOI 10.1897/04-131R.1

Ferreira NGC, Morgado R, Santos MJG, Soares AMVM, Loureiro S (2015). Biomarkers and energy reserves in the isopod *Porcellionides pruinosus*: The effects of long-term exposure to dimethoate. Science of the Total Environment 502:91-102. DOI: 10.1016/j.scitotenv.2014.08.062

Fischer E, Farkas S, Hornung E, Past T (1997) Sublethal effects of an organophosphorous insecticide, dimethoate, on the isopod *Porcellio scaber* Latr. Comparative Biochemistry and Physiology 116C: 161-166. DOI 10.1016/s0742-8413(96)00164-8

Hornung E, Farkas S, Fischer E (1998) Tests on the isopod *Porcellio scaber*. In: Løkke H, van Gestel CAM (Eds) Handbook of Soil Invertebrate Toxicity Tests. John Wiley & Sons, Chichester, 207–226.

Jänsch S, Garcia M, Römbke J (2005) Acute and chronic isopod testing using tropical *Porcellionides pruinosus* and three model pesticides. European Journal of Soil Biology 41: 143-152. DOI 10.1016/j.ejsobi.2005.09.010

Jemec A, Drobne D, Remškar M, Sepčić K, Tišler T (2008) Effects of ingested nano-sized titanium dioxide on terrestrial isopods (*Porcellio scaber*). Environmental Toxicology and Chemistry 27: 1904–1914. DOI 10.1897/08-036.1

Knigge T, Köhler HR (2000) Lead impact on nutrition, energy reserves, respiration and stress protein (Hsp 70) level in *Porcellio scaber* (Isopoda) populations differently preconditioned in their habitats. Environmental Pollution 108: 209-217. DOI 10.1016/S0269-7491(99)00188-8

Kolar L, Erzen NK, Hogerwerf L, Van Gestel CAM (2008) Toxicity of abamectin and doramectin to soil invertebrates. Environmental Pollution 151:182-189. DOI: 10.1016/j.envpol.2007.02.011

Kolar L, Jemec A, van Gestel CAM, Valant J, Hrzenjak R, Kozuh Erzen N, Zidar P (2010) Toxicity of abamectin to the terrestrial isopod *Porcellio scaber* (Isopoda, Crustacea). Ecotoxicology 19: 917-927. DOI 10.1007/s10646-010-0473-4

Lavtizar V, Berggren K, Trebse P, Kraak MHS, Verweij RA, Van Gestel CAM (2016) Comparative ecotoxicity of chlorantraniliprole to non-target soil invertebrates. Chemosphere 159:473-479. DOI: 10.1016/j.chemosphere.2016.06.036

Lemos MFL, van Gestel CAM, Soares AMVM (2009) Endocrine disruption in a terrestrial isopod under exposure to bisphenol A and vinclozolin. Journal of Soils and Sediments 9: 492–500. DOI 10.1007/s11368-009-0104-y

Lemos MFL, van Gestel CAM, Soares AMVM (2010a) Reproductive toxicity of the endocrine disrupters vinclozolin and bisphenol A in the terrestrial isopod *Porcellio scaber* (Latreille, 1804). Chemosphere 78: 907-913. DOI 10.1016/j.chemosphere.2009.10.063

Lemos MFL, Esteves AC, Samyn B, Timperman I, van Beeumen J, Correia A, van Gestel CAM, Soares AMVM (2010b) Protein differential expression induced by endocrine disrupting compounds in a terrestrial isopod. Chemosphere 79: 570-576. DOI 10.1016/j.chemosphere.2010.01.055

Lemos MFL, van Gestel CAM, Soares AMVM (2010c) Developmental toxicity of endocrine disrupters Bisphenol A and Vinclozolin in a terrestrial isopod. Archives of Environmental Contamination and Toxicology 59: 274-281. DOI 10.1007/s00244-010-9474-9

Loureiro S, Soares AMVM, Nogueira AJA (2005) Terrestrial avoidance behaviour tests as screening tool to assess soil contamination. Environmental Pollution 138: 121-131. DOI 10.1016/j.envpol.2005.02.013

Loureiro S, Santos C, Pinto G, Costa A, Monteiro M, Nogueira AJA, Soares AMVM (2006) Toxicity assessment of two soils from Jales Mine (Portugal) using plants: growth and biochemical parameters. Archives of Environmental Contamination and Toxicology 50: 182-190. DOI: 10.1007/s00244-004-0261-3

Mazzei V, Giannetto A, Brundo MV, Maisano M, Ferrante M, Copat C, Mauceri A, Longo G (2015) Metallothioneins and heat shock proteins 70 in *Armadillidium vulgare* (Isopoda, Oniscidea) exposed to cadmium and lead. Ecotoxicology and Environmental Safety 116:99-106. DOI: 10.1016/j.ecoenv.2015.03.007

Morgado RG, Gomes PAD, Ferreira NGC, Cardoso DN, Santos MJG, Soares AMVM, Loureiro S (2016) Toxicity interaction between chlorpyrifos, mancozeb and soil moisture to the terrestrial isopod *Porcellionides pruinosus*. Chemosphere 144:1845-1853. DOI: 10.1016/j.chemosphere.2015.10.034

Ribeiro S, Guilhermino L, Sousa JP, Soares AMVM (1999) Novel bioassay based on acetylcholinesterase and lactate dehydrogenase activities to evaluate the toxicity of chemicals to soil isopods. Ecotoxicology and Environmental Safety 44: 287-293. DOI: 10.1006/eesa.1999.1837

Ribeiro S, Sousa JP, Nogueira AJA, Soares AMVM (2001). Effect of endosulfan and parathion on energy reserves and physiological parameters of the terrestrial isopod *Porcellio dilatatus*. Ecotoxicology and Environmental Safety 49:131-138. DOI: 10.1006/eesa.2001.2045

Santos MJG, Soares AMVM, Loureiro S (2010) Joint effects of three plant protection products to the terrestrial isopod *Porcellionides pruinosus* and the collembolan *Folsomia candida*. Chemosphere 80: 1021–1030. DOI: 10.1016/j.chemosphere.2010.05.031

Silva PV, Silva ARR, Mendo S, Loureiro S (2014). Toxicity of tributyltin (TBT) to terrestrial organisms and its species sensitivity distribution. Science of the Total Environment 466:1037-1046. DOI: 10.1016/j.scitotenv.2013.08.002

Tourinho PS, Van Gestel CAM, Lofts S, Soares AMVM, Loureiro S (2013) Influence of soil pH on the toxicity of zinc oxide nanoparticles to the terrestrial isopod *Porcellionides pruinosus*. Environmental Toxicology and Chemistry 32: 2808–2815. DOI 10.1002/etc.2369

Tourinho PS, Van Gestel CAM, Jurkschat K, Soares AMVM, Loureiro S (2015a) Effects of soil and dietary exposures to Ag nanoparticles and AgNO_3_ in the terrestrial isopod *Porcellionides pruinosus*. Environmental Pollution 205:170-177. DOI: 10.1016/j.envpol.2015.05.044

Tourinho PS, Waalewijn-Kool PL, Zantkuijl I, Jurkschat K, Svendsen C, Soares AMVM, Loureiro S, Van Gestel CAM (2015b) CeO_2_ nanoparticles induce no changes in phenanthrene toxicity to the soil organisms *Porcellionides pruinosus* and *Folsomia candida*. Ecotoxicology and Environmental Safety 113:201-206. DOI: 10.1016/j.ecoenv.2014.12.006

Van Brummelen TC, van Gestel CAM, Verweij RA (1996). Long-term toxicity of five polycyclic aromatic hydrocarbons for the terrestrial isopods *Oniscus asellus* and *Porcellio scaber*. Environmental Toxicology and Chemistry 15: 1199-1210. DOI 10.1897/1551-5028(1996)015<1199:SCLTTO>2.3.CO;2

Van Ommen Kloeke AEE, Jager T, Van Gestel CAM, Ellers J, Van Pomeren M, Krommenhoek T, Styrishave B, Hansen M, Roelofs D (2012) Time-related survival effects of two gluconasturtiin hydrolysis products on the terrestrial isopod *Porcellio scaber*. Chemosphere 89:1084-1090. DOI: 10.1016/j.chemosphere.2012.05.074

Van Straalen NM, Donker M, Vijver MG, van Gestel CAM (2005) Bioavailability of contaminants estimated from uptake rates into soil invertebrates. Environmental Pollution 136: 409-417. DOI 10.1016/j.envpol.2005.01.019

Zidar P, Bozic J, Strus J (2005) Behavioral response in the terrestrial isopod *Porcellio scaber* (Crustacea) offered a choice of uncontaminated and cadmium-contaminated food. Ecotoxicology 14: 493-502. DOI: 10.1007/s10646-005-0005-9

Zidar P, van Gestel CAM, Strus J (2009) Single and joint effects of Zn and Cd on *Porcellio scaber* (Crustacea, Isopoda) exposed to artificially contaminated food. Ecotoxicology and Environmental Safety *72*: 2075-2082. DOI:10.1016/j.ecoenv.2009.06.009

Žižek S, Zidar P (2013) Toxicity of the ionophore antibiotic lasalocid to soil-dwelling invertebrates: Avoidance tests in comparison to classic sublethal tests. Chemosphere 92: 570-575. DOI: 10.1016/j.chemosphere.2013.04.007
